# Evaluation of Rheumatic Diseases Affecting the Temporomandibular Joint: A Cone Beam Computed Tomography Study and Literature Review

**DOI:** 10.3390/diagnostics14010004

**Published:** 2023-12-19

**Authors:** Zeliha Merve Semerci, Sevcihan Günen Yılmaz

**Affiliations:** Department of Oral and Maxillofacial Radiology, Faculty of Dentistry, Akdeniz University, Antalya 07070, Turkey; dentistsevcihan@hotmail.com

**Keywords:** temporomandibular joint, cone beam computed tomography, rheumatic diseases

## Abstract

Introduction: Due to the silent manifestation of temporomandibular joint (TMJ), dentists and rheumatologists may neglect treatment for this joint. Aims: The aim of this study was to investigate the TMJ components in patients with various rheumatic diseases and to compare them with a control group based on cone beam computed tomography (CBCT) images. Materials and Methods: This study comprised an assessment of the CBCT images of 65 patients (130 temporomandibular joints) with various rheumatic diseases (mostly rheumatoid arthritis) affecting the TMJ. Moreover, 65 patients (130 temporomandibular joints) with a similar age and gender distribution were examined as the control group. Pathologies were classified into a total of 12 types for the presence of any osseous changes in the condylar head or articular fossa or for joint space narrowing. Statistical analysis of all data was performed with SPSS version 18. The conformity of continuous variables to a normal distribution was examined by the Kolmogorov–Smirnov test. The Mann–Whitney U test was used to compare the means of two independent groups. The Pearson Chi-square test, Yates correction and Fisher’s exact test were used in the analysis of categorical variables. Results: The mean age of the patient and control groups was 50 ± 13 and 48 ± 16, respectively, and no statistically significant difference was found between the patient and control groups in terms of age distribution (*p* = 0.123). Condylar erosion, condylar flattening, subcondylar sclerosis, osteophytes, subcortical cysts, articular eminence resorption and articular eminence flattening rates were found to be statistically significantly higher in the patient group than in the control group (*p* < 0.05). Conclusions: Dentomaxillofacial radiologists should examine the bony components of the TMJ in patients with rheumatic diseases, and a multidisciplinary approach involving a dental specialist and rheumatologist is required.

## 1. Introduction

The temporomandibular joint (TMJ) is a ginglymoarthrodial joint that comprises the mandibular condyle, temporal bone and articular disc, and it is considered one of the most complex joints in the body [1]. Despite the fact that temporomandibular disorders (TMD) are typically associated with orofacial or dental concerns, we should classify intracapsular TMJ disease as arthropathy. The examination of the TMJ is often overlooked during the clinical evaluation of rheumatic patients, even though TMJ involvement has been reported for several rheumatic diseases [2,3]. Prior to investigating orofacial pain, dental complications and occlusal imbalances, underlying arthropathy should be considered in the presence of significant articular disc damage and signs of inflammation [4]. Likewise, dentists and rheumatologists should evaluate patients with rheumatic disease for TMJ involvement.

Rheumatic diseases refer to a group of conditions with a complex pathophysiology affecting multiple organs. Among these various diseases, inflammatory rheumatic arthritic diseases may show some systemic anomalies along with deformities in the synovial joints. In previous studies, it was mentioned that the TMJ is affected in rheumatic diseases such as rheumatoid arthritis (RA), ankylosing spondylitis (AS), juvenile idiopathic arthritis (JIA), systemic sclerosis (SS)/scleroderma, systemic lupus erythematosus (SLE), psoriatic arthritis (PA) and osteoarthritis (OA) [5].

The first principle in understanding how to manage TMD in rheumatic diseases is simply the conception that TMD may be the appearance of an undiagnosed or advanced rheumatic condition. Patients who experience pain on palpation of the peripheral joints are more likely to have soreness on palpation of the TMJ [5]. Clinical findings include sounds, morning stiffness, pain and limitations particularly in the lateral movement of the TMJ. The presence of changes in the TMJ is often neglected, specifically when treatment is focused on other joints. The treatment and monitoring of the temporomandibular joint is extremely important because of the movement impairment and the complexity of the rheumatic disease [6].

Rheumatic diseases affecting the TMJ present a diagnostic challenge to the dentist in the initial stages of the disease, because the symptoms, radiographic findings and laboratory markers are not unequivocal. Clinical assessment still remains a basic step, but a comprehensive radiological evaluation is necessary to confirm the clinical findings in severe cases. There may be a time delay between the appearance of symptoms and radiographic changes, so the absence of pathology on the radiograph does not exclude a possible disease [7].

Radiographic findings of osseous changes in the TMJ can be seen as osteophyte formation, flattening of the mandibular head, cortical erosion, a decrease in joint space, cortical irregularities, bird beak deformity of the condylar head and subcortical cysts [8]. The diagnostic imaging techniques used for rheumatic diseases of the TMJ include panoramic radiography, lateral radiographs, computed tomography (CT), cone beam computed tomography (CBCT), magnetic resonance imaging (MRI), arthrography and arthrotomography [9].

The osseous components of the joints are usually assessed using panoramic radiography and computed tomography, whereas the soft-tissue components are usually assessed using MRI. Ultrasonography is also particularly used for the assessment of the disk and soft tissues. It has been found that CT is superior to plain radiographs and magnetic resonance in detecting early degenerative changes in the mandibular condylar cartilage. For the radiologic diagnosis of degenerative osseous changes of the mandibular condylar cartilage, the reliability is excellent for CBCT [10,11]. Cone beam CT provides high-resolution multiplanar and three-dimensional (3D) images and delivers a substantially lower radiation dose and a shorter exposure time, and it ensures a lower cost compared to multislice CT. CBCT allows the examination of the TMJ anatomy without superimposition or distortion in all sagittal, coronal and axial slices. This technique is easy to perform, is reproducible and delivers a relatively low dose to the patient [11].

Thus, the objective of the current study was to compare osseous changes of the TMJ on CBCT images between patients with various rheumatic diseases affecting the TMJ and healthy controls.

## 2. Materials and Methods

This retrospective study was approved by the Research Ethics Board of Akdeniz University in Antalya, Turkey (70904504-66). All study procedures were performed in full compliance with the principles of the Declaration of Helsinki and comparable ethical standards.

The CBCT images of 65 patients (40 females, 25 males) who had various rheumatic diseases and no clinical complaints or signs and symptoms of TMD, based on their medical records, and a control group consisting of 65 age- and gender-matched patients (34 females, 31 males) who also had no clinical complaints or signs and symptoms of TMD or rheumatic disease but required other dental treatment necessitating CBCT radiographic imaging—such as pre-surgical planning for the removal of impacted teeth, dental implant planning, paranasal sinus imaging or other various dental treatments—were included in the study. These patients were referred to the Department of Oral and Maxillofacial Radiology, Faculty of Dentistry of Akdeniz University, between November 2021 and December 2022. To ensure image standardization and evaluation optimization, only images with a region of interest of 7 × 15 cm were employed. Informed consent was obtained from all individual participants included in the study.

Patients were excluded if they had a previous history of TMJ treatment or surgery, had undergone orthodontic treatment, had a previous history of trauma to the jaws, had TMJ pain associated with orofacial pain disorder, had neurological/neuropathic, endocrine or immune/autoimmune diseases with widespread pain, had a previous history of radiation treatment to the head and neck or there were inadequate existing clinical and CBCT data.

All CBCT images were acquired by the same X-ray technician and using the Veraview X800 (Morita, Kyoto, Japan) with a tube voltage of 100 kV, a tube current of 5 mA, an exposure time of 17.86 s and a voxel size of 0.125 mm.

The region of interest was 7 × 15 cm to cover the TMJ with the inferior orbital margin as the upper limit. During scanning, the patient position was set according to the manufacturer instructions, marked by a laser beam with the machine. Another vertical laser beam was aligned 4 cm in front of the condyle. Patients were instructed to swallow and bite on the bite block. The patient’s head was supported as recommended by the manufacturer, using a forehead support and chin rest. In order to evaluate the condylar position in the fossa and joint space, all examinations were performed in a central occlusal relationship according to the technique described in the study of Tsiklakis et al. [12].

A series of axial views of 1-mm thickness was automatically produced following the reconstruction of the raw data. TMJs were evaluated from reconstructed lateral slices perpendicular to the long axis of the condyle, coronal slices parallel to the long axis and central lateral image of the joint. To avoid misinterpretation, osseous changes had to be found in at least two consecutive slices. All images were evaluated by exporting to Digital Imaging and Communications in Medicine (DICOM) format. Images were viewed using a Dell monitor (22″ Full HD 1920 × 1080 display) in a dimly lit room [13,14].

There is no available radiographic scoring method to measure and evaluate TMJ changes in RA, as with Larsen’s classification, which is used to evaluate other individual joints; therefore, TMJ changes were evaluated as radiographic osteoarthritic features based on DC/TMD [15,16].

The two observers were asked to evaluate the following imaging characteristics relating to osseous changes of the condyles and in the articular fossa: (1) flattening, defined as a flat osseous contour deviating from the convex form; (2) erosion, defined as an area of decreased density of the cortical bone and the adjacent subcortical bone; (3) osteophytes, defined as marginal hypertrophy with sclerotic borders of the bony tissue arising from the surface of the condyle; (4) subcortical sclerosis, defined as an area of increased density of the cortical plate extending towards the bone marrow; (5) a subchondral cyst, defined as a small cavity underneath the articular surface that diverges from the normal bone marrow; (6) a bifid condyle; (7) loose joint bodies, defined as calcified structures that are discontinuous with the soft tissue or osseous structures of the joint; (8) joint space narrowing, defined as a reduction in space (<1.5 mm) in all anterior, superior and posterior directions; (9) increased joint space, defined as when the distance between the condylar head and mandibular fossa was more than 4 mm; (10) flattening of the articular eminence; (11) resorption in the articular eminence; and (12) ankylosis, defined as bony contact between the mandibular condyle and mandibular fossa [13,14,16].

### Statistical Analyses

The statistical analysis of all data was performed using IBM SPSS Statistics 18© Copyright SPSS Inc. Chicago, IL, USA, 1989, 2010. The conformity of continuous variables to a normal distribution was examined by the Kolmogorov–Smirnov test. Categorical variables in the study are presented as a frequency (*n*) and percentage (%). Continuous variables are presented with mean ± standard deviation (SD) and median (IQR 25–75) values. Since parametric test assumptions were not provided, the Mann–Whitney U test was used to compare the means of two independent groups. The Pearson Chi-square test, Yates correction and Fisher’s exact test were used in the analysis of categorical variables. The agreement between the two observations was examined with the Kappa statistic. The statistical significance level was accepted as 0.05 in the study.

## 3. Results

The mean age of the sample was 48 years (range: 39–61 years). Table 1 shows the distribution of the sample according to age and gender.

A total number of 260 TMJs (130 individuals) were evaluated by two observers blinded to the clinical characteristics of the patients and controls. Age, gender and osseous changes were recorded for each patient. Of the 130 participants, 65 were selected as the patient group and 65 as the age- and gender-matched control group.

**Figure 1:**
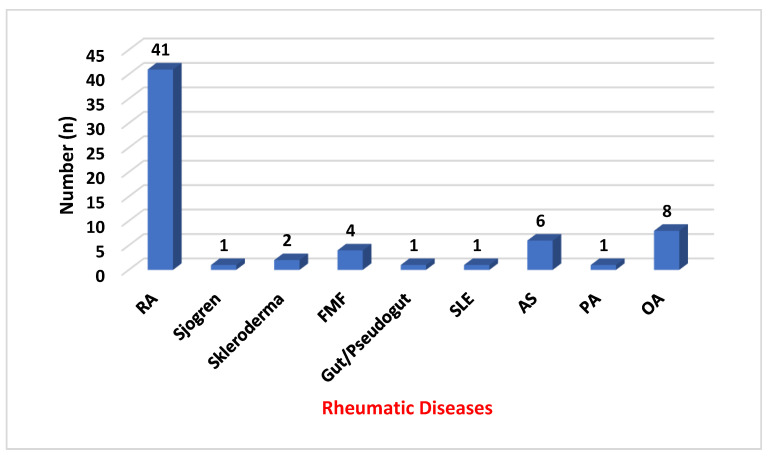
Distribution of diseases in the patient group.

The rheumatic disease distribution of the 65 patients in the patient group was as follows: RA 41, osteoarthritis (OA) 8, familial mediterranean fever (FMF) 4, scleroderma 2, Sjögren’s syndrome 1, systemic lupus erythematosus (SLE) 1, psoriatic arthritis (PA) 1. Figure 1 shows the distribution of diseases in the patient group.

Overall, 45.4% of the patients were male; this rate was 38.5% in the patient group and 52.3% in the control group, and the difference was statistically significant (*p* = 0.025). The median age was calculated as 50 (41–61) years in the patient group, 47 (34–57) years in the control group and 48 (39–61) years in the whole group. In terms of age distribution, the patient and control groups were similar (*p* = 0.123) (Table 1).

Of the 130 TMJs included in the patient group, 69 (53.1%) showed condylar flattening, 54 (41.5%) showed subcondylar sclerosis, 44 (33.8%) showed subcortical cysts, 3 (2.3%) showed bifid condyles, 21 (16.2%) showed articular eminence resorption, 6 (4.6%) showed loose joint bodies, 50 (38.5%) showed osteophytes, 5 (3.8%) showed ankylosis and 25 (19.2%) showed joint space narrowing in both the first and second observer evaluations. In the patient group, an increased joint space was not observed in any TMJs in both observer evaluations.

In accordance with the first observer, condylar flattening was determined in 49 (37.7%) TMJs in the patient group, while the second observer determined condylar flattening in 48 (36.9%) TMJs. Similarly, while the first observer detected articular fossa flattening in 10 (7.7%) TMJs, the second observer detected articular eminence flattening in 11 TMJs (8.5%).

Analyzing the temporomandibular joint osseous changes in the patient and control groups, condylar erosion, condylar flattening, subcondylar sclerosis, osteophytes, subcortical cysts, the resorption of articular eminence and the flattening of articular eminence rates were found to be statistically significantly higher in the patient group compared to the control group, both in the evaluations of the first observer and the second observer (*p* < 0.05). Bifid condyles, loose joint bodies, ankylosis, joint space narrowing and increased joint spaces were found to be statistically similar in both the first and second observer evaluations in the patient and control groups (*p* > 0.05). When the compatibility of the evaluations of the first observer and the second observer was analyzed with the Kappa statistic, it was determined that all evaluations were statistically significantly compatible (*p* < 0.001). A very high level of agreement was found for the values of condylar erosion (κ = 0.97), condylar flattening (κ = 0.98), subcondylar sclerosis (κ = 0.99), osteophytes (κ = 0.99), flattening of articular eminence (κ = 0.95) and joint space narrowing (κ = 0.98). In the evaluations of subcortical cysts, bifid condyles, the resorption of articular eminence, loose joint bodies, ankylosis and an increased joint space, there was complete agreement (κ = 1) (Table 2).

Concordance was rated using the criteria of Landis and Koch (1977): 0.01–0.205 slight; 0.21–0.405 acceptable; 0.41–0.605 moderate; 0.61–0.805 considerable; and 0.81–1.005 almost perfect [17].

**Figure 2:**
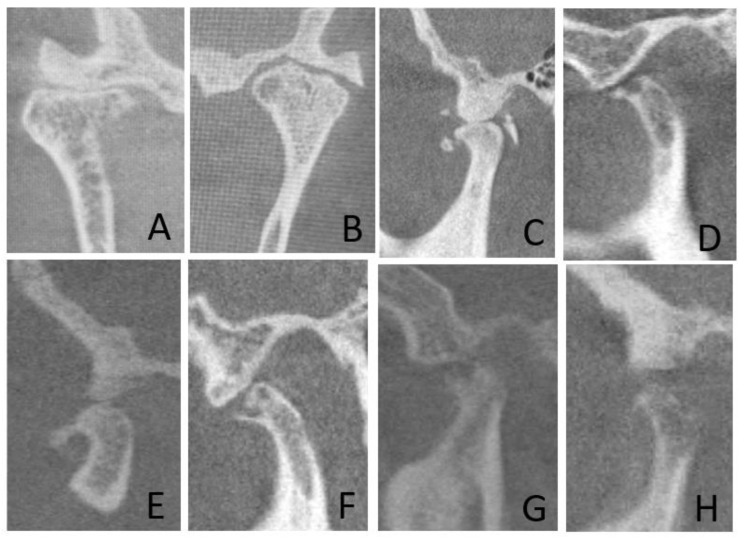
Examples of the CBCT images of osseous changes in the temporomandibular joints (TMJs). (**A**)—flattening of the condyle, subcortical sclerosis and ankylosis in a male with rheumatoid arthritis; (**B**)—joint space narrowing, subcortical cyst formation, condylar surface and articular eminence resorption in a female with scleroderma; (**C**)—loose calcified bodies in a male with rheumatoid arthritis; (**D**)—subcortical cyst formation in a female with psoriatic arthritis; (**E**)—flattening of the condyle and osteophyte formation in a male with osteoarthritis; (**F**)—multiple subcortical cysts in a female with rheumatoid arthritis; (**G**)—progressive condyle erosion in a male with pseudogout; (**H**)—articular eminence and condyle resorption in a female with SLE.

**Figure 3:**
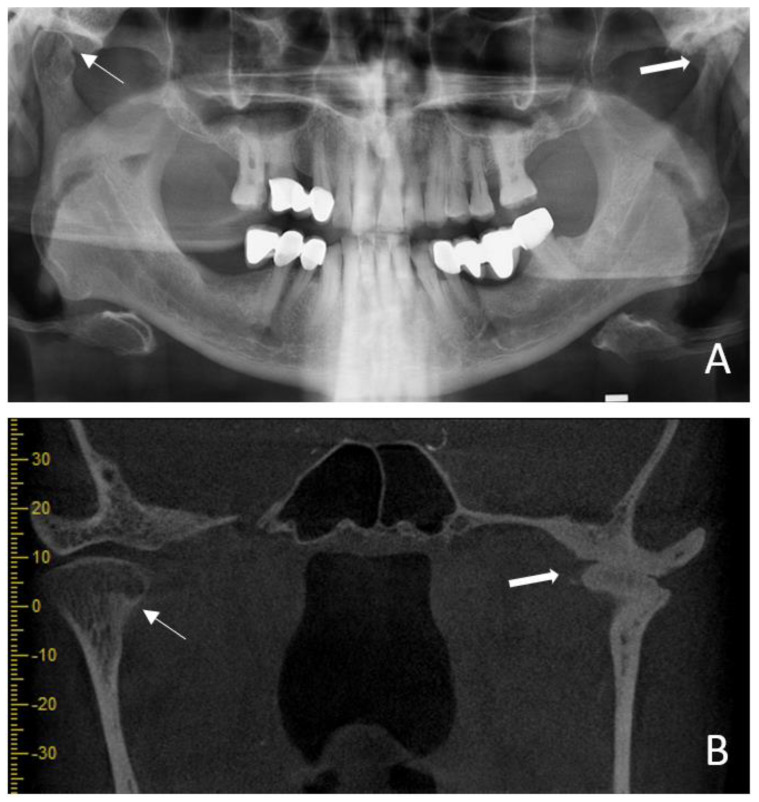
(**A**) presents the panoramic radiographic image of a 62–year–old patient diagnosed with rheumatoid arthritis for the past 12 years. Resorption at the anterior part of the mandibular condyle (indicated by a thin white arrow), multiple radiopacities at the condylar head, condylar flattening and a decrease in the joint space (indicated by a thick white arrow) are observed. (**B**) displays the coronal cone beam computed tomography (CBCT) image of the same patient. Resorption at the anterior part of the mandibular condyle (indicated by a thin white arrow), multiple radiopacities at the condylar head and ankylosis (indicated by a thick white arrow) are observed. The findings are consistent with those observed in panoramic radiography, although the actual extent of the disease can be better determined.

Exemplary images of osseous changes in the TMJs are shown in Figure 2 and Figure 3.

## 4. Discussion

Rheumatologic diseases can significantly impact the TMJ, causing a range of symptoms and complications. Conditions such as rheumatoid arthritis, systemic lupus erythematosus and psoriatic arthritis are known to affect the TMJ, leading to inflammation, pain and restricted jaw movement [5].

It has been discovered that the prevalence of TMJ involvement in patients with rheumatic disease varies substantially depending on the diagnostic criteria, the population investigated and the methods used to evaluate the TMJ. There are limited studies regarding TMJ radiographic evaluation in patients with rheumatic diseases, especially in PA, systemic scleroderma and AS. Previous studies have focused on the TMJ radiographic findings of RA, while knowledge of the nature of TMJ involvement in other rheumatic diseases is still limited and further research in this field is necessary. Erosions and subcortical cysts of the mandibular condyle are typical initial radiological findings [18].

RA is a systemic, symmetrical, peripheral, inflammatory polyarticular connective tissue disease caused by erosive synovitis, resulting in joint deformity and instability. The prevalence of RA is 1%, affecting women more than men in a 3:1 ratio and an age range between 35 and 45 years [19]. The TMJ is often affected in RA, particularly in its severe form. The incidence of its involvement ranges from 5% to 86% [19]. In addition, a correlation between the laboratory values of various inflammatory markers related to rheumatoid arthritis and the progression of TMD has been reported. Correlations have also been reported to exist between the number of swollen joints, rheumatoid factor (RF), sedimentation rate (ESR), C-reactive protein (CRP), thrombocyte count and plasma tumor necrosis factor alpha levels and temporomandibular joint involvement. According to these studies, TMJ involvement is more prevalent in severe RA patients [20,21]. Ankylosing spondylitis (AS) is a chronic, systemic, inflammatory disease that primarily affects the axial skeleton. TMJ involvement in patients with AS varies between 4% and 35%. Psoriatic arthropathy is an inflammatory seronegative arthritis that affects 5–8% of patients with psoriasis [22]. Half the patients with psoriatic arthropathy have TMJ symptoms, and up to 90% have signs of dysfunction. Involvement of the TMJ is more common and more severe in patients with psoriatic arthropathy than in those with uncomplicated psoriasis and healthy individuals [23]. Systemic sclerosis, also called scleroderma, is a multisystem connective tissue disease with an unknown etiology, and it is defined by infection and fibrotic and vascular changes in the skin and internal organs [24]. Chebbi et al. assessed the effect of systemic sclerosis on TMD. They reported that the early diagnosis of TMD in systemic sclerosis patients is necessary [25]. They observed that the assessment and knowledge of the oral and dental changes due to scleroderma are necessary for dentists because they may lead to the earlier diagnosis of systemic sclerosis [26]. Lupus erythematosus is a chronic autoimmune disease that affects various organs, including joints [27]. Jonsson et al. reported that TMD is common in lupus erythematosus patients [28]. In their study, Crincoli et al. included fifty-five patients diagnosed with systemic lupus erythematosus (SLE), comprising 9 men and 46 women. They compared the temporomandibular disorder (TMD) symptoms in these patients with healthy controls based on the DC/TMD criteria. The results revealed that SLE patients reported a higher frequency (95.8%) of oral and TMJ symptoms, including dysgeusia, stomatodynia, masticatory muscle pain during function, neck and shoulder muscle pain and the presence of tinnitus. These results underscore the heightened prevalence of TMD-related symptoms in individuals with systemic lupus erythematosus, shedding light on the intricate relationship between SLE and temporomandibular joint dysfunction [29].

In the literature, there are studies investigating the impact of rheumatic diseases and inflammatory biomarkers, as well as disease severity, on TMJ involvement [20,30]. In a study conducted by Yılmaz et al., which involved 28 rheumatoid arthritis (RA) patients and 29 control subjects, the progression of TMJ and masticatory muscle involvement was examined using Disease Activity Score 28 (DAS28) scoring, magnetic resonance imaging (MRI) and lateral panoramic radiography. The TMJ symptoms were identified as frequent findings and were associated with the mean duration of the disease in RA. The study suggests that laboratory findings should be taken into consideration when assessing disease-activity-related TMJ involvement [30].

In our study, we observed that the most common temporomandibular joint osseous pathologies in individuals with rheumatic diseases were condylar erosion at a rate of 53%, subcondylar sclerosis at a rate of 41%, osteophytes at a rate of 39% and condylar flattening at a rate of 38%, respectively.

Although the rheumatic disease of the temporomandibular joint can be confused with the findings of a degenerative joint disease due to the advanced age of the patient group, we believe that the effect of this on our results was minimal, as the difference between the findings of the control group and the patient group was statistically significant and our results were consistent with the previous literature [31,32]. Subcortical sclerosis of the condylar surface or fossa is considered a variation, especially with regard to advanced age, remodeling or the association with mandibular hyperfunction as an attempt at adaptation. However, the manifestation of generalized sclerosis of the subchondral bone is associated with joint degradation and may be a result of the presence of TMJ rheumatic disease [33]. Degenerative bone changes, such as the presence of erosion and flattening in the mandibular condyle, are usually noticed in CT scans 5 to 10 years after the onset of symptoms [34]. Helenius et al. examined temporomandibular joint pathologies in patients with various rheumatic diseases, using panoramic and lateral panoramic radiographs, and they observed distinct erosions in 17% of patients with rheumatoid arthritis, 19% of patients with mixed connective tissue disease and 38% of patients with spondyloarthritis. Larheim et al. evaluated 36 patients, 28 of whom were symptomatic, with various rheumatic diseases (mostly rheumatoid arthritis), and found that 25 temporomandibular joints showed TMJ pathology in the CT results [35]. In the study of Wenneberg et al., radiographic changes were found significantly more often in subjects with rheumatoid arthritis (66%), psoriatic arthritis (38%) and ankylosing spondylitis (30%) than in controls (12%). For this reason, they reached the conclusion that rheumatoid arthritis is a more severe disease than psoriatic arthritis or ankylosing arthritis regarding temporomandibular joint involvement [31]. In another study by Wenneberg et al., they used panoramic radiography to compare 90 patients with AS with age- and sex-matched controls. Radiographic changes were observed in 25% of patients and 11% of controls [22]. The first aim of management is to relieve pain. Initial conservative measures include jaw resting, physiotherapy, non-steroidal anti-inflammatory drugs (NSAIDs) and occlusal splints. Around 80% of patients will have their symptoms resolved by conservative treatments alone [36].

Medical specialists that regularly provide treatment for temporomandibular joint disorder in patients without rheumatic conditions are familiar with the predictable pattern of disease advancement that aligns with the Wilkes categorization. Wilkes made a noteworthy observation regarding the significant association between the temporal progression of the biological lesions and various clinical and radiological results, which exhibited a strong correlation [37]. In the context of rheumatic diseases, the temporal development of temporomandibular disorders may exhibit unforeseen patterns. It is important to note that a direct association between clinical observations and radiographic results should not be directly presumed. A multidisciplinary approach is crucial, combining the expertise of rheumatologists, dentists and other healthcare professionals. Rheumatologists play a pivotal role in addressing the systemic aspects of these diseases, managing inflammation and prescribing appropriate medications. Dentists, particularly those specializing in oral and maxillofacial medicine, focus on localized TMJ symptoms, offering treatments such as occlusal splints, physical therapy and, in severe cases, surgical interventions [5]. The collaboration between these disciplines ensures a holistic approach, considering both the systemic and local manifestations of rheumatologic diseases affecting the TMJ, ultimately improving patient outcomes and quality of life.

The clinical examination of TMJ rheumatic disease is insufficient to fully evaluate the osseous and soft-tissue changes in the mandibular condyle, and the need for TMJ imaging is usually determined after a thorough anamnesis and clinical examination. Cone beam computed tomography scans are widely regarded as a valuable imaging method for the viewing of the temporomandibular joint (TMJ) compared to other techniques. Panoramic radiography has several limitations, such as structural distortion, superimposition from the zygomatic process and the inability to show the entire articular surface of the TMJ [38].

Ahmad et al. mentioned in their study that panoramic radiographs also have low reliability and low sensitivity in detecting osseous changes in the TMJ [16].

The lateral radiographs show too much overlap of other anatomical structures, and, in addition, the soft tissues are not visible. Axial tomography provides a sufficient view of the erosions and osteophytes on the surface of the condyle, but their interpretation is difficult. Computed tomography (CT) is valid for the imaging of the mandibular condyle but the device has a high cost and a relatively high radiation dose, and there is poor access to equipment, all of which limits its use for the evaluation of the TMJ [39].

MRI is considered the prime imaging method to evaluate the soft-tissue components of the temporomandibular joint in circumstances when the diagnosis of a soft-tissue pathology is uncertain or when ionizing radiation should be avoided [40]. In conventional MRI, soft tissues typically appear bright on the images, making it challenging to distinguish between different structures, especially when they have similar signal intensities. With advancements in imaging methods, there are studies indicating that MRI can be used to depict the bony components of the TMJ. These methodologies are often known as ultra-short echo time (UTE) and zero echo time (ZTE) sequences. These approaches have demonstrated the capacity to facilitate valuable bony tissue imaging using MRI. However, CBCT or MDCT remains the gold standard for TMJ bony components [29].

Although CBCT is unable to display actual Hounsfield units, which can provide a more valid quantitative assessment of bone density, it has higher sensitivity with regard to viewing the morphology of the osseous components of the joint, cortical bone continuity, subcortical bone destruction and sclerosis. CBCT is better at detecting changes in condylar and articular eminence flattening, osteophyte formation and erosion [41].

CBCT is also superior to CT in analyzing lateral slices in isolation and combining coronal and lateral slices [41]. Therefore, CBCT was the imaging method used in this study.

According to the study of Librizzi et al., in which two different cone beam computed tomography devices were used for the detection of osseous changes in the TMJ, osseous changes in the TMJ could be better differentiated in smaller fields of view (FOV). For this reason, we only included images in 7 × 15 cm FOVs in our study [42].

This study was limited by being a monocenter study and the fact that the CBCT findings were surveyed retrospectively. All patients were referred because of TMD, which included a wide range of symptoms; this may explain the high rate of radiographic degeneration of temporomandibular joint bony structures in the age- and sex-matched control group.

Another limitation of this study was that the clinical evaluation of the patients could not be assessed because it was planned retrospectively. Moreover, there was a limited number of patients with rheumatic diseases other than rheumatoid arthritis; therefore, the group of patients could not be compared among themselves. For future studies, the abovementioned limitations can be overcome with larger patient and control groups, with the aim of helping them to achieve better quality of life.

## 5. Conclusions

Due to the silent manifestation of TMJ disorders, dentists and rheumatologists may neglect treatment for this joint. A multidisciplinary approach is essential to reduce the physical and psychological consequences of rheumatic diseases affecting the TMJ.

Considering the study results, individuals with rheumatic diseases, even asymptomatic patients, are susceptible to osteoarthritic pathologies in the temporomandibular joint. The assessment of TMJ bony components for osteoarthritic changes in individuals with various rheumatic diseases can be conducted through cone beam computed tomography. To facilitate treatment, early diagnosis and disease prevention, clinicians are advised to employ CBCT imaging when deemed necessary.

## Figures and Tables

**Table 1:** Demographic characteristics in patient and control groups.

Variables	Patient Group (*n* = 130)	Control Group (*n* = 130)	Total (*n* = 260)	*p*
Gender, *n* (%)				
Male	50 (38.5%)	68 (52.3%)	118 (45.4%)	0.025 *
Female	80 (61.5%)	62 (47.7%)	142 (54.6%)	
Age (years)				
Mean ± SD	50 ± 13	48 ± 16	49 ± 15	0.123 ^µ^
Median (IQR)	50 (41–61)	47 (34–57)	48 (39–61)	

* Pearson Chi-square test, ^µ^ Mann–Whitney U test.

**Table 2:** The osseous pathologies of the temporomandibular joint in the patient and control groups and the agreement between observers. Numbers 1 and 2 represent Observer-1 and Observer-2 evaluations, respectively.

Variables	Patient Group (*n* = 130)	Control Group (*n* = 130)	Variables	*p* ^1^	Kappa	*p* ^2^
Condylar erosion-1	69 (53.1%)	41 (31.5%)	110 (42.3%)	<0.001	0.97	<0.001
Condylar erosion-2	69 (53.1%)	38 (29.2%)	107 (41.2%)	<0.001		
Condylar flattening-1	49 (37.7%)	24 (18.5%)	73 (28.1%)	0.001	0.98	<0.001
Condylar flattening-2	48 (36.9%)	23 (17.7%)	71 (27.3%)	0.001		
Subcortical sclerosis-1	54 (41.5%)	17 (13.1%)	71 (27.3%)	<0.001	0.99	<0.001
Subcortical sclerosis-2	54 (41.5%)	18 (13.8%)	72 (27.7%)	<0.001		
Osteophyte-1	50 (38.5%)	22 (16.9%)	72 (27.7%)	<0.001	0.99	<0.001
Osteophyte-2	50 (38.5%)	21 (16.2%)	71 (27.3%)	<0.001		
Subcortical cyst-1	44 (33.8%)	17 (13.1%)	61 (23.5%)	<0.001	1	<0.001
Subcortical cyst-2	44 (33.8%)	17 (13.1%)	61 (23.5%)	<0.001		
Bifid condyle-1	3 (2.3%)	9 (6.9%)	12 (4.6%)	0.139	1	<0.001
Bifid condyle-2	3 (2.3%)	9 (6.9%)	12 (4.6%)	0.139		
Articular eminence resorption-1	21 (16.2%)	4 (3.1%)	25 (9.6%)	0.001	1	<0.001
Articular eminence resorption-2	21 (16.2%)	4 (3.1%)	25 (9.6%)	0.001		
Articular eminence flattening-1	10 (7.7%)	1 (0.8%)	11 (4.2%)	0.014	0.95	<0.001
Articular eminence flattening-2	11 (8.5%)	1 (0.8%)	12 (4.6%)	0.008		
Loose joint bodies-1	6 (4.6%)	2 (1.5%)	8 (3.1%)	0.281	1	<0.001
Loose joint bodies-2	6 (4.6%)	2 (1.5%)	8 (3.1%)	0.281		
Ankylosis-1	5 (3.8%)	3 (2.3%)	8 (3.1%)	0.722	1	<0.001
Ankylosis-2	5 (3.8%)	3 (2.3%)	8 (3.1%)	0.722		
Joint space narrowing-1	25 (19.2%)	15 (11.5%)	40 (15.4%)	0.122	0.98	<0.001
Joint space narrowing-2	25 (19.2%)	14 (10.8%)	39 (15%)	0.082		
Increased joint space-1	0 (0%)	1 (0.8%)	1 (0.4%)	0.999	1	<0.001
Increased joint space-2	0 (0%)	1 (0.8%)	1 (0.4%)	0.999		

^1^ Pearson Chi-square test, Yates correction, Fisher’s exact test. ^2^ Kappa statistic.

## Data Availability

Reported results are unavailable due to privacy restrictions.

## Abbreviations

TMJ	Temporomandibular joint
CBCT	Cone beam computed tomography
TMD	Temporomandibular disorders
RA	Rheumatoid arthritis
AS	Ankylosing spondylitis
JIA	Juvenile idiopathic arthritis
SS	Systemic sclerosis
SLE	Systemic lupus erythematosus
PA	Psoriatic arthritis
OA	Osteoarthritis
CT	Computed tomography
MRI	Magnetic resonance imaging
RF	Rheumatoid factor
ESR	Sedimentation rate
CRP	C-reactive protein
DICOM	Digital Imaging and Communications in Medicine
MDCT	Multidetector Computed Tomography
